# Risk Preferences in Surrogate Decision Making

**DOI:** 10.1027/1618-3169/a000371

**Published:** 2017-09-18

**Authors:** Eleonore Batteux, Eamonn Ferguson, Richard J. Tunney

**Affiliations:** ^1^ School of Psychology, University of Nottingham, UK

**Keywords:** surrogate decisions, risk preferences, probability discounting, decision making

## Abstract

**Abstract.** There is growing evidence that decisions made on behalf of other people differ from the decisions we make for ourselves because we are less affected by the subjective experience of their outcome. As a result, the decisions we make for other people can be more optimal. This experiment investigated surrogate decision making using a probability discounting task where participants made choices between risky and sure options. Psychological distance between the decision maker and the recipient was manipulated by having participants make decisions for themselves, their friend, and another unknown participant. Risk preferences were closer to neutrality (i.e., more consistent with expected value) when making decisions on behalf of another participant than when making decisions for themselves or a friend. We conclude that subjective risk preferences are attenuated in surrogate decision making. Findings are discussed in relation to inconsistencies in the literature and theories of surrogate decision making.

A large proportion of our everyday decisions are made on behalf of other people (Tunney & Ziegler, 2015). In such cases we act as a surrogate decision maker by making a decision of which the outcome will impact another person – the recipient of the decision. Recent models of surrogate decision making have attempted to uncover the underlying psychological processes that explain why we often make decisions for other people that we would not make for ourselves. The following experiment investigated how people’s risk preferences differ when making surrogate decisions as opposed to their own and why that may be the case. Although this question has already been posed, the literature concerning surrogate risk preferences presents inconsistencies which render its answer unclear. We report an experiment that differs from similar work in that the decisions that were made had a real outcome affecting real recipients.

The literature regarding self-other differences in risk preferences is often contradictory. A substantial amount suggests that people exhibit less risk aversion when making decisions for other people than when making decisions for themselves. This has been found using relationship scenarios (Beisswanger, Stone, Hupp, & Allgaier, 2003; Stone & Allgaier, 2008; Stone, Choi, de Bruin, & Mandel, 2013; Wray & Stone, 2005) as well as monetary decision tasks such as choices between gambles (Chakravarty, Harrison, Haruvy, & Rutström, 2011; Mengarelli, Moretti, Faralla, Vindras, & Sirigu, 2014; Polman, 2012; Ziegler & Tunney, 2015). However, there have also been reports using similar monetary decision tasks that surrogate decisions accentuate risk aversion (Pahlke, Strasser, & Vieider, 2015; Reynolds, Joseph, & Sherwood, 2011) and others that report no self-other differences (Stone, Yates, & Caruthers, 2002). Benjamin and Robbins (2007) also report an absence of self-other differences using the Balloon Analog Risk Task (BART). Using the same investment task, Pollmann, Potters, and Trautmann (2014) found that risk-taking increased whereas Eriksen and Kvaløy (2010) found that risk-taking decreased when making decisions for others. Using a similar task, Kvaløy, Eriksen, and Luzuriaga (2014) identified that risk-averse participants took more risks on behalf of another person, whereas risk-seeking participants took fewer risks. These inconsistencies do not seem to be attributable to the type of task used. Are there other methodological differences which might be informative?

In terms of differences between studies which investigated real and hypothetical decisions, a clear pattern does not emerge either. Studies which found that risk aversion decreases in surrogate decisions, in the psychology literature, used hypothetical decisions for a friend (Beisswanger et al., 2003; Stone & Allgaier, 2008; Stone et al., 2013; Wray & Stone, 2005) or for a stranger (Ziegler & Tunney, 2015), and in the economic literature used real decisions for a stranger (Chakravarty et al., 2011; Kvaløy et al., 2014; Mengarelli et al., 2014; Polman, 2012; Pollmann et al., 2014). Studies which found that risk aversion increases in surrogate decisions used real decisions for a stranger (Kvaløy et al., 2014), for the participant and a stranger (Pahlke, Strasser, & Vieider, 2015), or for a group (Reynolds et al., 2011). Studies which found an absence of difference used hypothetical decisions for a friend (Benjamin & Robbins, 2007; Stone et al., 2002) or real decisions for a stranger (Stone et al., 2002). From this it is also difficult to assess the impact of the identity of the recipient, although real scenarios where the decision maker knew the recipient were not investigated. In the present experiment, participants made real decisions for themselves, for a friend and for another unknown participant to test whether the identity of the recipient affects the outcome of the decision. Why do we expect these self-other differences in risk preferences?

Findings suggesting that subjective risk preferences are attenuated in surrogate decisions are consistent with the risk-as-feelings hypothesis (Loewenstein, Weber, Hsee, & Welch, 2001). The hypothesis posits that risk preferences are the result of emotional reactions to risk, rather than a purely cognitive evaluation of risk. Given that a surrogate decision maker is not the recipient of their decision, it follows that an empathy gap would emerge between them and the outcome (Loewenstein, 1996). Assuming the presence of this empathy gap, the risk-as-feelings hypothesis predicts that when making surrogate decisions people are less influenced by emotional reactions to risk and therefore exhibit risk preferences that are closer to risk neutrality.

There is increasing evidence that self-other differences in decision making can be explained by a tendency toward less emotional bias when making decisions on behalf of other people. Indeed, surrogate decisions have been found to be more optimal than the decisions people make for themselves. In delay-discounting tasks, people tend to favor the immediate reward over the larger delayed reward more often for themselves than for others (Charlton et al., 2013; Kim, Schnall, & White, 2013; Pronin, Olivola, & Kennedy, 2008; Ziegler & Tunney, 2012). This could be the result of reduced subjective experience of the immediate reward when one is not the recipient of the decision. It could also be explained by reduced sensitivity to the uncertainty associated with choosing the delayed reward, in accordance with the evidence suggesting that people have different attitudes toward risk when making decisions on behalf of other people.

Furthermore, the decision maker is affected by the outcome differently than the recipient and is likely to adopt a more reasoned approach to the decision process, thereby reducing their emotional involvement. According to construal-level theory, psychological distance between the decision maker and the recipient of the decision leads to more abstract thinking (Trope & Liberman, 2010), meaning that the recipient’s immediate subjective experience of the outcome is less likely to be taken into account when making decisions on their behalf. We therefore expect subjective risk preferences to become increasingly attenuated as psychological distance between the decision maker and the recipient increases. Are these predictions supported by current theories and models of surrogate decision making? We will consider the contributions of Social Values Theory (Stone & Allgaier, 2008) and the Egocentric Anchoring and Adjustment model (Epley, Keysar, Van Boven, & Gilovich, 2004) and show how Tunney and Ziegler’s model (2015) provides a more comprehensive framework for addressing self-other differences.

Social Values Theory (Stone & Allgaier, 2008) proposes that in the domain of risk, self-other differences in decision making will arise when there is a social value placed on risk. Based on their previous research which failed to find self-other differences in monetary decisions (Stone et al., 2002), they conclude that there is no social value placed on risk in monetary decisions. Given the amount of evidence suggesting that there are such self-other differences, this conclusion is difficult to believe. It also seems unlikely that all decisions made on behalf of other people are based solely on social values and ignore factors such as the identity of the recipient. In fact, most of the evidence supporting Social Values Theory is based on differences between decisions people make for themselves and a friend (Beisswanger et al., 2003; Stone & Allgaier, 2008; Stone et al., 2002, 2013). A different pattern of self-other differences may appear when the recipient is not a friend.

The Egocentric Anchoring and Adjustment model (Epley et al., 2004) suggests that when adopting another’s perspective, people use their own as an anchor and adjust from it. The level of adjustment accounts for differences between the decision maker and the recipient and stops once a plausible estimate is reached. Surrogate decisions are therefore egocentrically biased. Ziegler and Tunney (2012) conducted a delay-discounting study where participants made decisions for themselves and a variety of recipients who varied in psychological distance from them (i.e., varied in degree of relatedness) and found that choices made for others varied systematically from choices made for the self as psychological distance increased, which is what we predict in this experiment. The model assumes that decisions are based on adopting the recipient’s perspective, however there might be cases where the decision-maker wishes to make a decision based on what they want or on what they think is in the recipient’s best interest.

Tunney and Ziegler’s (2015) model of surrogate decision making assumes that the decision maker engages in perspective-taking which varies according to the features of the surrogate decision. They could consider what is in the recipient’s best interest (benevolent perspective), what they would do if they were the recipient (projected perspective), and try to simulate what the recipient would choose (simulated perspective). They may also make a decision that serves their own interest irrespective of the wishes of the recipient (egocentric perspective). The decision maker compares the outcomes of different perspectives and computes a subjective utility estimation, which is distorted by a number of biasing factors. The significance of the decision and accountability of the decision maker are expected to play a role. The relationship between the decision maker and the recipient is also predicted to impact the decision process: familiarity and similarity with the recipient will influence the decision maker’s ability to engage in simulated perspective-taking, while higher proximity and closeness between the decision maker and the recipient will increase the decision maker’s emotional involvement in the decision process. Designs which differ in terms of the significance of the decision, accountability of the decision maker, and identity of the recipient are likely to find distinct patterns in self-other differences, which could explain some of the inconsistencies in the literature concerning risk.

The present experiment used a probability discounting task, which involves choosing between a guaranteed reward and a chance of winning another reward. Participants often discount the probabilistic reward in favor of a guaranteed reward of lower expected value: the subjective value of a reward decreases when its occurrence is probabilistic. As the rate of discounting is faster for higher than lower probabilities, probability discounting is best described by a hyperbolic curve representing the subjective value of the probabilistic reward as a function of the probability of obtaining the reward (Rachlin, Raineri, & Cross, 1991). As stated above there is evidence that delay-discounting rates are reduced in surrogate decision making, but there is no evidence as of yet that probability discounting rates are also reduced. Given that discounting the probabilistic option is considered risk-averse, we predict that discount rates will decrease systematically as psychological distance between the decision maker and the recipient increases. Participants’ compensation was made contingent on their decisions as well as those that others made for them so that participants knew that they were making real decisions. Although other studies have reported little or no consequence of using real versus hypothetical rewards in probability discounting (Hinvest & Anderson, 2010; Matusiewicz, Carter, Landes, & Yi, 2013), the data remains equivocal and has not been investigated in surrogate decision making.

## Method

### Design

A within-subjects design was used where participants made decisions for themselves, their friend, and another unknown participant. The independent variable was the recipient of the decisions and the dependent variable was the probability discount rates. The order of presentation of each recipient was counterbalanced across participants.

### Ethics Statement

Ethics approval was obtained from the Ethics Committee at the University of Nottingham, reference 808.

### Participants

Participants (*n* = 110) were recruited in pairs from the University of Nottingham. Participants were asked to come in with a friend who would be taking part in the experiment as well. Although the level of friendship was not controlled for, because participants were recruited in pairs, some level of familiarity was assumed. The age and gender of participants within each pair were not controlled for. The age group ranged from 18 to 44 years (*M* = 22.9, *SD* = 3.43). There were 49 males and 61 females.

### Probability Discounting Task

The probability discounting task was performed on a computer using PsychoPy (Peirce, 2007). The task consisted of three conditions: participants made decisions either for themselves, for their friend, or for another participant. All trials consisted of making a choice between a sure option and a probabilistic option using the “up” and “down” arrow keys. The trials were presented in blocks of probabilities, from the highest to the lowest, within which the sure options were presented in descending order. There were 11 sure options (£95, £85, £75, £65, £55, £45, £35, £25, £15, £10, £5) and 7 sets of probabilities of winning £100 (95%, 90%, 70%, 50%, 30%, 10%, 5%). Each sure option was presented with each probabilistic option once. All 77 trials were presented in the same order in each condition. There was no deadline for responding.

### Participant Compensation

A participant’s compensation was the sum of the outcomes of three choices: a choice that participant made for themselves, a choice their friend made for them, and a choice the previous participant made for them. Only a proportion of that sum was received and was set so that compensations were on average £3. Participants were told that one of the trials in each condition was real – a proportion of its outcome would constitute part of its recipient’s compensation. The real trial was always the choice between “£45 for sure” and a “50% chance of winning £100” as both options have similar expected values. To minimize reciprocation between participants and their friend, they were not given a breakdown of their compensation.

### Procedure

Pairs of participants were tested at the same time, in the same room, on separate computers. Communication between the two participants was not permitted. The experimenter remained in the room throughout. Once the probability discounting task was completed by both participants, the experimenter prepared their compensation. If the probabilistic option was chosen on a probe trial, its outcome was computer generated.

## Results

The indifference points for each probability and recipient (self, friend, other) were generated for every participant. The indifference point is the value at which the participant is equally likely to choose the sure option and the probabilistic option – the participant’s subjective value of the probability. The average of the sure values immediately before and after participants switched from choosing the sure option to the probabilistic option was taken as the indifference point. If participants always selected the probabilistic option, £95 was considered the indifference point; if they always selected the sure option, £5 was considered the indifference point.

In the case where for any given probability participants crossed over from one option to the other twice, the average of the two indifference points was taken as their indifference point. If they crossed over more than twice, an indifference point was not generated. A few participants switched from the probabilistic option to the sure option rather than the other way around and were excluded from the entire analysis on suspicion that they did not understand the task. When trials switched to the next probability participants sometimes selected the probabilistic option instead of the sure option, presumably by accident; in that case, indifference points were not generated. One participant was excluded for making stereotyped responses (i.e., always pressing the same button) on suspicion that they did not understand the task. Participants who were still missing more than two indifference points for a given recipient were excluded from the entire analysis as a reliable discount curve could not be fitted to their data.

Discount rates were estimated for the remaining participants (*n* = 99). Probability discounting is best described by a hyperbolic discount curve^1^ (Rachlin et al., 1991) where the stated probability (*p*) is transformed to the odds against winning (θ), where θ = (1/*p*) − 1. The subjective discounted value (*v*) of an amount (*V*) is discounted as a function (*h*) of the odds against winning (θ) the amount (*V*):
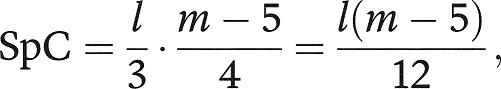
1


**Figure 1:**
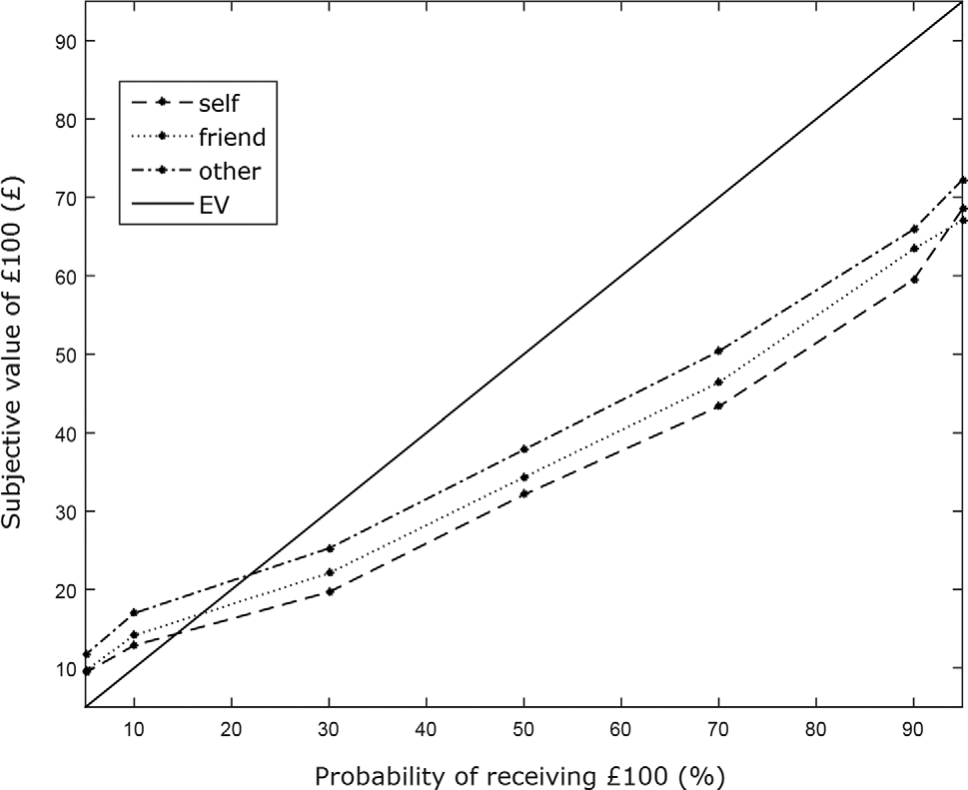
The average subjective values of £100 across participants as a function of the probability of receiving £100 for each recipient (self, friend, other), as well as the expected value (EV) of the probability of receiving £100. Subjective values that are lower than the expected value are considered risk-averse whereas subjective values that are higher than the expected value are considered risk-seeking.

The parameter *h* which describes discount rates across probabilities was estimated separately for every participant using a nonlinear regression. Participants had one value of *h* for each recipient. The *h* parameters were then log-transformed as they were highly positively skewed. Higher values of *h* indicate higher levels of discounting. The indifference points from which individual values of *h* were estimated are shown in Figure 1.

**Figure 2:**
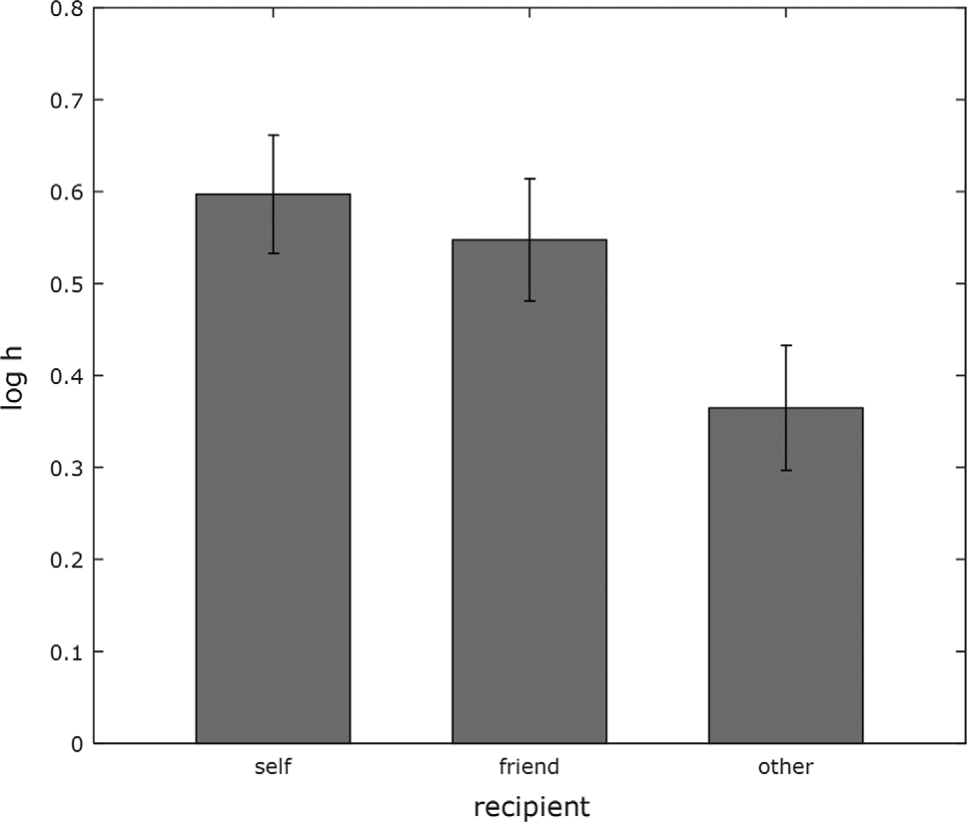
Discount rates for each recipient (self, friend, other) with error bars representing the standard error of the mean. Higher values of log *h* indicate higher levels of discounting or risk aversion.

The discount rates (log *h* values) for self (*M* = 0.6, *SD* = 0.64, CI [0.47, 0.72]) were higher than the discount rates for friend (*M* = 0.55, *SD* = 0.66, CI [0.42, 0.68]) and the discount rates for other (*M* = 0.36, *SD* = 0.67, CI [0.23, 0.5]) (see Figure 2). To test whether discount rates differed across recipients, the log *h* values were entered into a repeated-measures analysis of variance (ANOVA) with recipient as the within-subjects factor. Mauchly’s test indicated that the assumption of sphericity had been violated (*p* < .001), therefore degrees of freedom were corrected using the Greenhouse-Geisser method. There was a significant effect of recipient (*F*_1.67,163.692_ = 9.538, *MSE* = 0.186, *p* < .001, η_p_^2^ = 0.089). There was also a significant linear trend by recipient (*F*_1,98_ = 16.021, *MSE* = 0.167, *p* < .001, η_p_^2^ = 0.141). Paired-sample *t*-tests revealed that self-discount rates were significantly higher than other discount rates (*t*_98_ = 4.003, *p* < .001), that friend discount rates were significantly higher than other discount rates (*t*_98_ = 2.814, *p* = .006) but that self and friend were not significantly different from each other (*t*_98_ = 1.158, *p* = .250).

**Figure 3:**
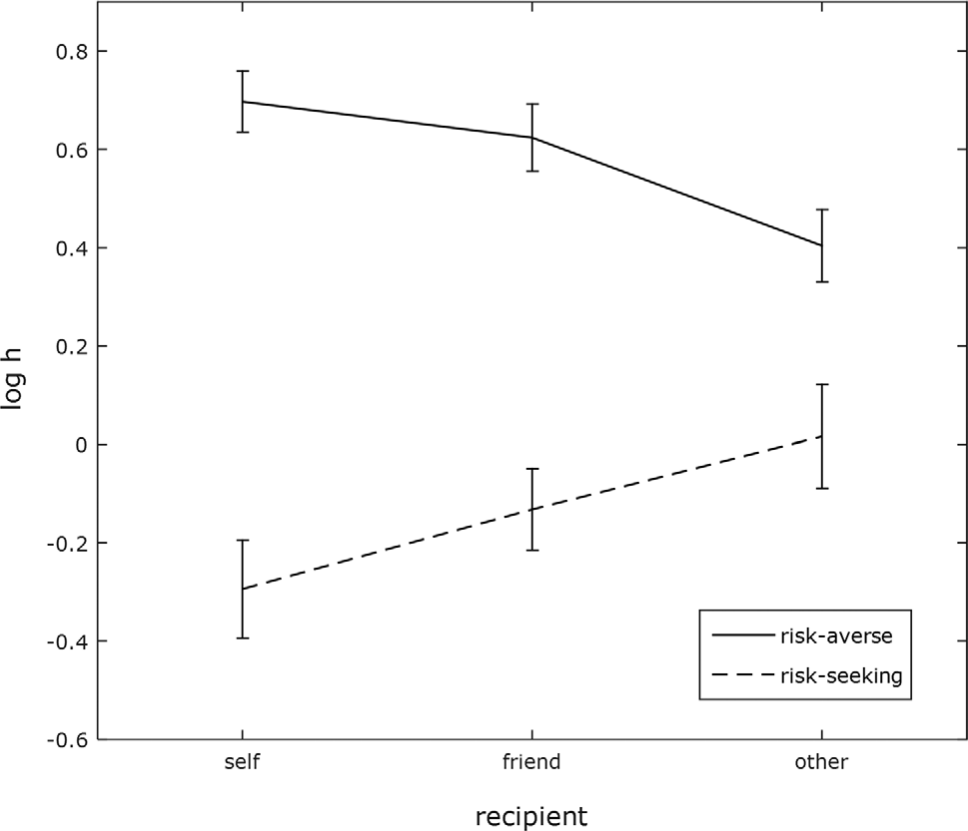
Discount rates of risk-averse versus risk-seeking participants for each recipient (self, friend, other) with error bars representing the standard error of the mean. A log *h* value of zero indicates risk-neutrality, above zero indicates risk-aversion, and below zero indicates risk-seeking.

Probability discounting assumes that people are risk-averse, as they are expected to discount the probabilistic option in favor of the sure option when the latter’s expected value is lower than the former’s. We therefore predicted that people will be less risk-averse when making decisions on behalf of other people. However, there were a proportion of participants (*n* = 10) who were risk-seeking for themselves rather than risk-averse (risk-seeking from an economic perspective, i.e., choosing a probabilistic option which has a lower expected value than the sure option). Given the risk-as-feelings hypothesis, such individuals would be expected to be less risk-seeking for other people than themselves, rather than less risk-averse. Indeed, risk-seeking participants show the opposite pattern to risk-averse participants (see Figure 3). Therefore it can be predicted that people make decisions on behalf of other people that are closer to risk neutrality than the decisions they make for themselves.

To test this prediction, a new dependent variable was generated to measure how far participant’s decisions deviated from risk neutrality (log *h* = 0). The discount rates for self (*M* = 0.66, *SD* = 0.58, CI [0.54, 0.77]) deviated more from risk neutrality than the discount rates for friend (*M* = 0.62, *SD* = 0.59, CI [0.5, 0.74]) and the discount rates for other (*M* = 0.54, *SD* = 0.54, CI [0.43, 0.65]). To test whether discount rates deviated from risk neutrality differently across recipients (self, friend, other), the new log *h* values were entered into a repeated-measures ANOVA with recipient as the within-subjects factor. There was a significant effect of recipient (*F*_2,196_ = 4.614, *MSE* = 0.075, *p* = .011, η_p_^2^ = 0.045). There was also a significant linear trend by recipient (*F*_1,98_ = 11.273, *MSE* = 0.059, *p* = .001, η_p_^2^ = 0.103). Paired-sample *t*-tests revealed that self-discount rates deviated significantly more from risk neutrality than other discount rates (*t*_98_ = 3.358, *p* = .001) but that there was no difference between friend and other (*t*_98_ = 1.883, *p* = .063) and between self and friend (*t*_98_ = 0.920, *p* = .360).

## Discussion

The results replicate previous findings showing that participants discount probabilistic options in favor of sure options more often than expected utility theory would predict (Rachlin et al., 1991). In accordance with prospect theory (Kahneman & Tversky, 1979), the indifference points show that participants underweighted large probabilities and overweighed small probabilities.

The key finding was differences in self-other decision making. Surrogate decisions became increasingly different from decisions made for the self as psychological distance between the decision maker and the recipient increased. As predicted, participants had shallower rates of discounting in surrogate decisions, meaning that the decisions they made for others were closer to those predicted by the expected value – arguably more economically rational. Therefore, consistent with a large proportion of previous findings, participants made more risk-averse decisions for themselves than for other people. However, around 10% of participants were risk-seeking when making decisions for themselves, meaning that they chose the probabilistic option over the sure option more often than expected utility theory would predict. Interestingly, these participants were less risk-seeking for others than themselves, or more risk-averse. They show self-other differences in the opposite direction as do risk-averse participants, although given the low frequency of such participants, statistical differences cannot be reliably computed. Nevertheless, participants’ decisions were closer to risk neutrality when making decisions for a stranger than when making decisions for themselves or for their friend. Taken together, these results suggest that people’s risk preferences are attenuated in surrogate decision making, as expected by the risk-as-feelings hypothesis (Loewenstein et al., 2001).

Participants made similar decisions for themselves and for their friends, but made decisions that were closer to risk neutrality on behalf of the other participant. People are likely to be more affected by the outcome of the decision they make on behalf of a friend than a stranger. In the case of someone who is risk-averse, it follows that they would not want to take more risks for a friend than they would take for themselves. However, when making a decision on behalf of a stranger, their decision process is less affected by the negative prospect of taking a risk that does not pay off if they consider the decision to be a reasonable assessment of risk. Similarly, Ziegler and Tunney (2012) found that surrogate decisions were less impulsive – or more rational – as psychological distance between the decision maker and the recipient increased, and that decisions for a friend were closest to decisions for the self while decisions for a stranger were the furthest.

A crucial difference between the friend and the other condition was that participants’ friends were sitting next to them during the experiment whereas they never crossed paths with the other participant. This spatial proximity will have further reduced psychological distance between the decision maker and their friend, which probably contributed to the absence of difference between decisions made for the self and decisions made for a friend. Perhaps if the other participant had been in the room as well there would have been less of a difference between self and other decisions, although studies have reported self-other differences while the recipient and the decision maker were in the same room (Eriksen & Kvaløy, 2010; Pahlke et al., 2015; Polman, 2012). Furthermore, the same trend was found by Ziegler and Tunney (2012) where participants made decisions for hypothetical others (including friend and stranger), therefore indicating that the results are not solely attributable to differences in spatial proximity. Nevertheless, whether the results are due to differences in relationship’s closeness or spatial proximity, they are both manifestations of psychological distance and support the suggested theoretical explanations of self-other differences in surrogate decision making (Tunney & Ziegler, 2015). The question remains as to whether the effects of psychological distance are indeed additive. The use of a within-subjects design could have accentuated the salience of self-other differences, although these differences have also been found in between-subject designs using real monetary decisions (Polman, 2012; Pollmann et al., 2014).

Similar results have been reported when comparing people’s decisions and their predictions of other people’s. Hsee and Weber (1997) found that participants were highly risk-averse when making decisions for themselves but predicted that other people would be less risk-averse. No difference was found when participants made predictions for someone who sat next to them. Faro and Rottenstreich (2006) report that people systematically predict others’ choices to be closer to risk neutrality than their own choices: when people are risk-averse they predict others to be less risk-averse, but when people are risk-seeking they predict others to be less risk-seeking. The difference between choices and predictions was substantially diminished when the other person was a close friend or when participants were told to put themselves “in the shoes” of the other person. Both papers interpret their findings in relation to the empathy gap, stating that an ability to empathize with the other person will increase the predictor’s dependence on his own risk preferences rather than risk neutrality to make a prediction. The findings we report here replicate these patterns, suggesting that people may make surrogate decisions in accordance with what they predict the recipient’s risk preferences to be.

In terms of interpreting these results relative to theories of surrogate decision making, Social Values Theory (Stone & Allgaier, 2008) is unhelpful as it does not predict self-other differences in monetary decision making. The Egocentric Anchoring and Adjustment model (Epley et al., 2004) can only account for the fact that decisions made on behalf of others deviated systematically from decisions made for the self as psychological distance between the decision maker and the recipient increased. Tunney and Ziegler’s (2015) model can explain self-other differences in terms of calibration – relationship between decision maker and recipient – and ability to empathize with the recipient. In fact, it would be useful to assess or manipulate the latter in future research. It is also possible that participants felt they would be held accountable by their friend for their decisions, which led them to be as risk-averse when making decisions for their friend as for themselves. Indeed, Pollmann et al. (2014) found that being held accountable mitigates self-other differences by increasing risk aversion in surrogate decisions. Given the evidence concerning predictions of others’ risk preferences, perhaps participants were making a simulated decision – in accordance with what they thought others’ risk preferences were. This would imply that people are not able to make an accurate simulated decision if on the one hand they rely on their own risk preferences when they can empathize with the recipient and on the other they revert to risk neutrality when they cannot empathize with the recipient. In the former case, they are in fact making a projected decision whereas in the latter, they are perhaps making a more benevolent decision.

Finally, the present findings help to explain some of the inconsistencies in the literature regarding self-other differences in risky decision making. Firstly, the identity of the recipient of the decision clearly has an impact on the decision process; concluding that there are no self-other differences in risky decision making from findings where the recipient was a friend would be misleading. Secondly, a failure to examine potential differences in surrogate decision making between people who tend to be risk-averse and people who tend to be risk-seeking would be responsible for an absence of self-other differences if they cancel each other out. Identifying whether a decision is risk-seeking or risk-averse is problematic however; it is debatable whether being indifferent between a sure option and a probabilistic option of equal expected value in probability discounting can be taken as risk neutrality. Lastly, it is possible that finding that people make less risk-seeking decisions on behalf of other people is the product of an experimental design which encourages participants to be risk-seeking for themselves.

### Conclusion

The present findings support that there exist self-other differences in surrogate decision making involving risk. Risk preferences are attenuated when making decisions for other people: risk-averse participants take more risks for others whereas risk-seeking participants take less. This is a valuable contribution to previous findings given that decisions had an outcome that affected real recipients and that there is no published research looking at probability discounting in surrogate decision making. To avoid further inconsistencies in the literature, the role the identity of the recipient has on the decision process should be more carefully considered. Self-other differences arose when making decisions for a stranger but not a friend, which suggests they vary depending on the identity of the recipient. To deepen our understanding of surrogate decision making, it would be interesting to investigate whether participants were actually engaging in simulated perspective-taking or if they were simply less biased toward their own risk preference when making their decision.

## Electronic Supplementary Material

The electronic supplementary material is available with the online version of the article at http://dx.doi.org/10.1027/1618-3169/a000371
*ESM 1*. Data (xls).
Indifference points and discount rates for each participant.

## Footnotes

1 There exists another mathematical description of probability discounting, the hyperboloid discount function, which has been found to be a better fit to the data (Green & Myerson, 2004). This version adds a free parameter *s* which is said to describe the nonlinear psychophysical scaling of amount (*V*) and/or odds against (θ): However, the parameter *h* describes the rate of discounting when *s* is held constant (Holt, Green, & Myerson, 2003) and there is evidence that in probability discounting *s* is not held constant and is amount-dependent, meaning that the psychophysical scaling interpretation of *s* is incorrect (McKerchar, Green, & Myerson, 2010; Myerson, Green, & Morris, 2011). Given that the interpretation of *s* is unclear and the aim here is to compare rates of discounting across conditions, *s* is not a useful parameter. Additionally, the parameter *s* is usually needed when different amounts (*V*) are used within the same experiment, whereas here the amount (*V*) was held constant. In light of this, the hyperbolic discount curve was used to compute values of *h*.
